# Design of a High-Speed Prosthetic Finger Driven by Peano-HASEL Actuators

**DOI:** 10.3389/frobt.2020.586216

**Published:** 2020-11-27

**Authors:** Zachary Yoder, Nicholas Kellaris, Christina Chase-Markopoulou, Devon Ricken, Shane K. Mitchell, Madison B. Emmett, Richard F. ff. Weir, Jacob Segil, Christoph Keplinger

**Affiliations:** ^1^Paul M. Rady Department of Mechanical Engineering, University of Colorado Boulder, Boulder, CO, United States; ^2^Materials Science and Engineering Program, University of Colorado Boulder, Boulder, CO, United States; ^3^Biomechatronics Development Laboratory, Rocky Mountain Regional VA Medical Center, Aurora, CO, United States; ^4^Engineering Plus Program, University of Colorado Boulder, Boulder, CO, United States

**Keywords:** prosthesis, prosthetic hand, HASEL, electrohydraulic actuator, soft robotics, bioinspired, modeling

## Abstract

Current designs of powered prosthetic limbs are limited by the nearly exclusive use of DC motor technology. Soft actuators promise new design freedom to create prosthetic limbs which more closely mimic intact neuromuscular systems and improve the capabilities of prosthetic users. This work evaluates the performance of a hydraulically amplified self-healing electrostatic (HASEL) soft actuator for use in a prosthetic hand. We compare a linearly-contracting HASEL actuator, termed a Peano-HASEL, to an existing actuator (DC motor) when driving a prosthetic finger like those utilized in multi-functional prosthetic hands. A kinematic model of the prosthetic finger is developed and validated, and is used to customize a prosthetic finger that is tuned to complement the force-strain characteristics of the Peano-HASEL actuators. An analytical model is used to inform the design of an improved Peano-HASEL actuator with the goal of increasing the fingertip pinch force of the prosthetic finger. When compared to a weight-matched DC motor actuator, the Peano-HASEL and custom finger is 10.6 times faster, has 11.1 times higher bandwidth, and consumes 8.7 times less electrical energy to grasp. It reaches 91% of the maximum range of motion of the original finger. However, the DC motor actuator produces 10 times the fingertip force at a relevant grip position. In this body of work, we present ways to further increase the force output of the Peano-HASEL driven prosthetic finger system, and discuss the significance of the unique properties of Peano-HASELs when applied to the field of upper-limb prosthetic design. This approach toward clinically-relevant actuator performance paired with a substantially different form-factor compared to DC motors presents new opportunities to advance the field of prosthetic limb design.

## Introduction

**Figure 1:**
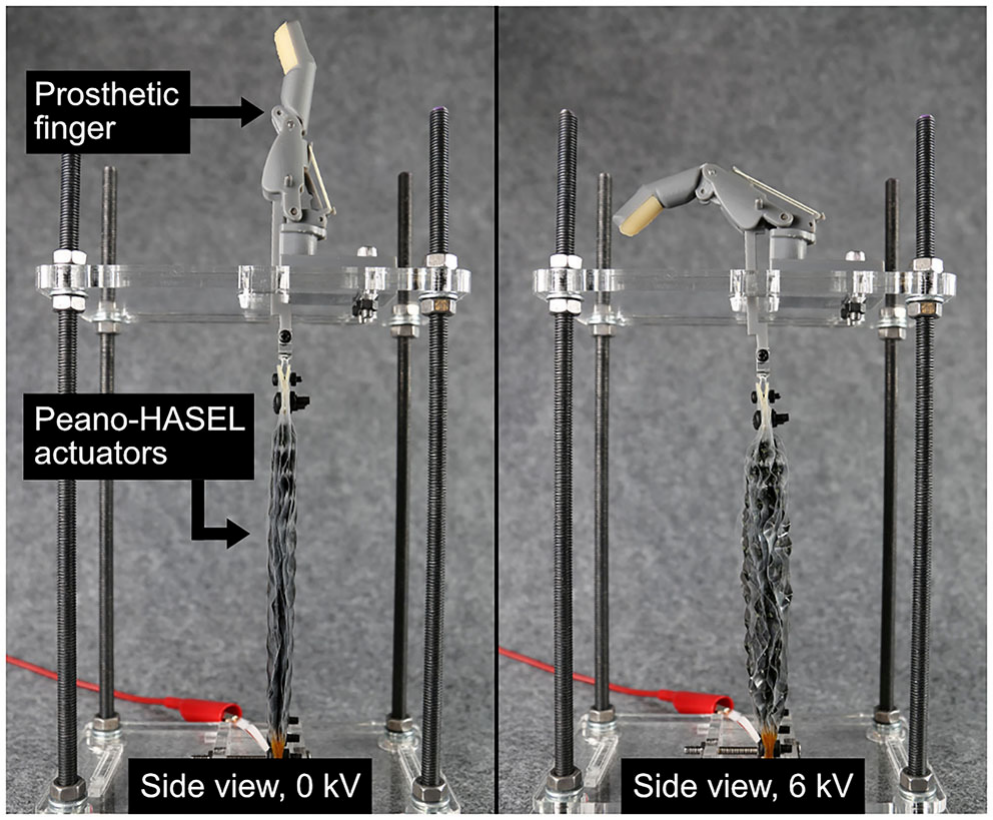
An array of Peano-HASEL actuators, a new type of high-performance, electrohydraulic artificial muscle, driving a custom prosthetic finger. The actuators linearly contract under an applied voltage of 6 kV, which causes flexion of the prosthetic finger.

The field of upper limb prosthetic design seeks to recreate what was lost after amputation. In order to accomplish this feat, prosthetic devices require compact, stable, and clinically robust materials which integrate actuation to interact with the external environment (Childress and Weir, 2004). These types of actuators are more important now, in light of recent progress across a wide range of related fields like neural interfaces and osseointegration (Ortiz-Catalan et al., 2014; Tan et al., 2015). However, a remnant actuator technology—the DC electric motor—has been used for generations and has constrained the design of prosthetic devices. Here we investigate if hydraulically amplified self-healing electrostatic (HASEL) actuators, a new type of high-performance artificial muscles, will be able to improve upon the existing DC motor to facilitate the creation of lifelike prosthetic limbs with enhanced functionality. The proposed design can be seen in Figure 1.

The first use of DC motors in prosthetic limbs occurred in the 1940s and 1950s (Childress, 1985). These systems required substantial development before an actuated prosthesis was able to withstand the rigors of everyday use. In 1968 at Northwestern University, the first self-contained, externally-powered proportional myoelectric prosthetic limb was fit by Childress and others (Childress, 1985). Since then, miniaturization of motors, their associated electronics, and an increase in battery energy density caused rapid development of single degree-of-freedom prehensors/hands and subsequently multi-functional hands (Childress, 1985; Childress and Weir, 2004; Belter et al., 2013).

The advantages of multi-functional prosthetic hands include: the ability to create unique grasps/postures, production of clinically-appropriate forces/speeds, and an anatomically appropriate structure with all five digits. However, the use of DC motors has also constrained the device in ways that have caused disappointment among upper limb amputees. Biddiss et al. outlined the design priorities among upper limb amputees and highlighted the primary concern among powered prosthetic hand users: distribution of weight (Biddiss et al., 2007). Other design priorities listed in the top ten among powered prosthetic hand users also directly relate to the use of DC motors including cost, heat, reliability, and size. In all cases, the use of DC motors in the distal elements of a prosthetic system causes numerous concerns among upper-limb amputees such as weight, heat, reliability, and mechanical compliance (Cordella et al., 2016). Many of these concerns have not been addressed even with updated releases of popular commercial hands because the same fundamental element (DC motor) is still being used.

In multifunctional prosthetic hands, the location of the motor defines the design of the prosthetic fingers and thumb. Commercially available devices integrate the motor in the digit (iLimb by Touch Bionics, Vincent by Vincent Systems) or the palm (Bebionic by RSL Steeper, Michelangelo by Otto Bock) (Belter et al., 2013). The location of the DC motor then informs the type of transmission necessary for the digit. Various transmission designs are used throughout the industry including planetary gearheads, spur gear trains, ball screws, and other custom devices (Belter et al., 2013); new approaches that integrate DC motors with soft transmissions have been explored in academia (O'Brien et al., 2018). Finally, the kinematic design of the digit translates the force/torque produced by the actuator to the force/displacement of the digit. Typical kinematic designs of the commercially available prosthetic fingers include two phalanges where the distal interphalangeal (DIP) joint is fixed, and the metacarpal phalangeal (MCP) and proximal interphalangeal (PIP) joints are free to rotate.

In several thorough reviews, (Cura et al., 2003; Biddiss and Chau, 2008; Controzzi et al., 2014) prototype actuators with varied principles of operation are studied for use in prosthetic hands. Pneumatics, hydraulics, shape memory allows, and dielectric elastomer actuators (DEAs) have been studied, but have not been translated into clinical solutions due to difficulties with their implementation as a stable clinical system. Biddiss and Chau (2008) detail the challenges and opportunities of DEAs for use in upper limb prostheses and highlight several outstanding challenges including: unreliable temporal control, insufficient force production, and lack of anthropomorphic size/weight. While numerous artificial muscles have been proposed or explored as actuators in prostheses and wearable assistive devices, all have pitfalls in either strength, speed, reliability, complexity, and/or controllability which impedes their use outside of a laboratory setting (Biddiss et al., 2007; Park et al., 2014; Wu et al., 2015). Therefore, traditional robotic components like gears and electric motors are still the preferred method of linear actuation for prosthetic devices. Biddiss and Chau's thorough review was published in 2008 with the request to reevaluate “soft actuators” in the future.

Over 10 years later, the field of soft actuators has dramatically advanced, as part of a larger push toward “soft robotics” (Kim et al., 2013). Soft robotics incorporates compliant, lightweight, and multifunctional components into machines to mimic the adaptability and robustness of biological organisms. Biomimetic actuators are seen as desirable since their compliance should translate to robustness toward external loading events during everyday use in the field. However, the capabilities of soft robots continue to be limited by the lack of soft actuators with sufficient all-around performance in areas such as force production, speed, and efficiency.

**Figure 2:**
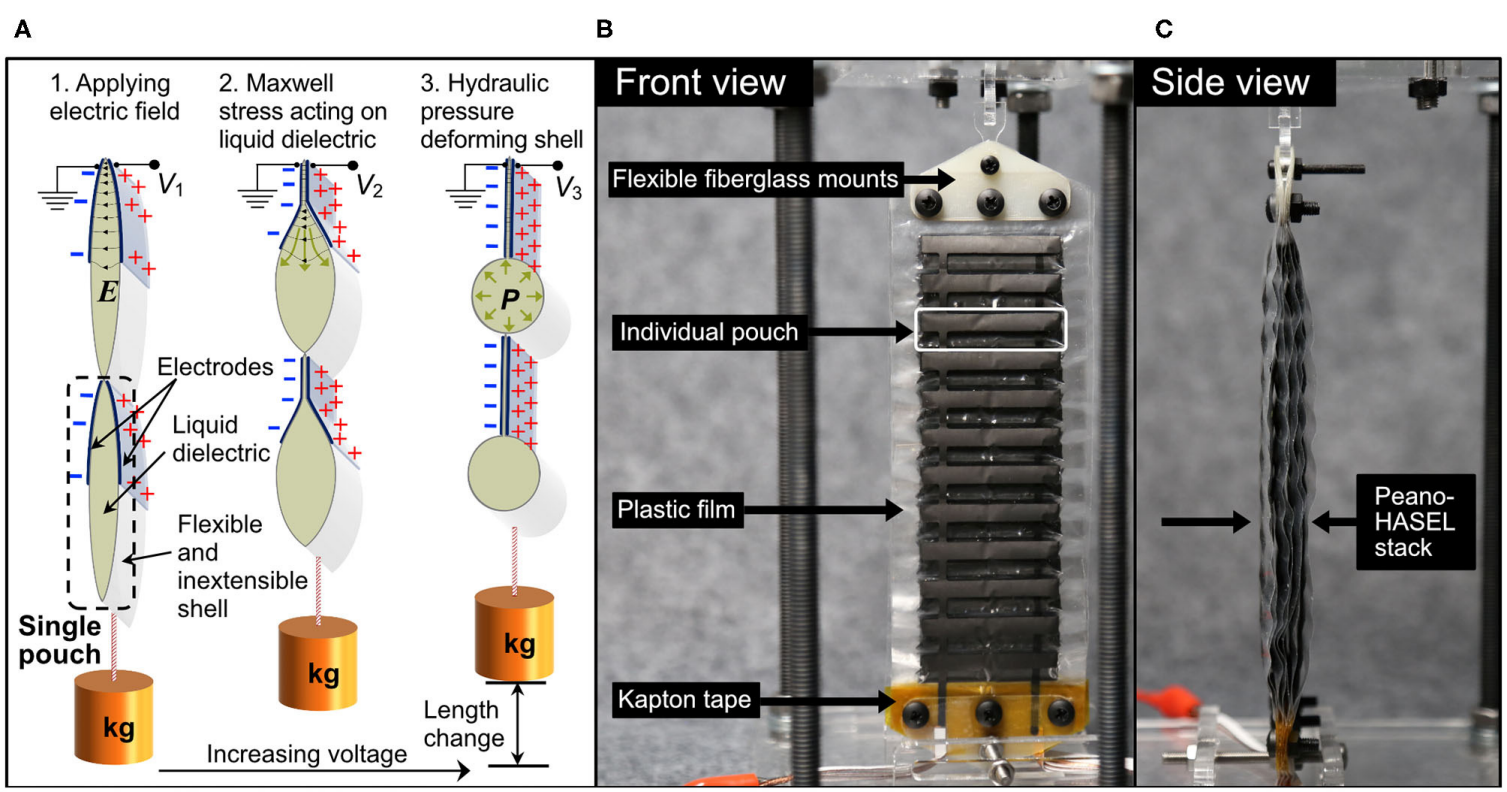
**(A)** Schematic of a Peano-HASEL actuator showing basic structure and principles of operation. When voltage is applied, the resulting electric field *E* draws the electrodes together and locally displaces the liquid dielectric. The resulting hydraulic pressure *P* deforms the inextensible thermoplastic shell and causes linear contraction. **(B,C)** Photos of a stack of 7 Peano-HASEL actuators, with ancillary components labeled.

A new class of soft actuators, or artificial muscles, termed hydraulically amplified self-healing electrostatic (HASEL) actuators (Acome et al., 2018; Kellaris et al., 2018; Mitchell et al., 2019; Wang et al., 2020), merge the design freedom of soft fluidic actuators (Polygerinos et al., 2017) with the biomimetic, muscle-like performance and portability of dielectric elastomer actuators (Anderson et al., 2012; Koh et al., 2017; Duduta et al., 2019; Ji et al., 2019). One type of HASEL, termed the Peano-HASEL actuator, is depicted in Figure 2 (Kellaris et al., 2018). The actuator consists of a flexible, yet inextensible polymer shell which is filled with a liquid dielectric and partially covered by electrodes. When a voltage is applied across the electrodes, an electrostatic force causes the electrodes to controllably zip together, thereby forcing the fluid into the volume of the shell which is not covered by the electrodes. This local displacement of the fluid causes the cross-section of the uncovered portion of the shell to change from a flatter cross-section to a more circular one. Since the shell is inextensible, this shape change results in a linear contraction of the actuator (Kellaris et al., 2018). Peano-HASEL actuators offer advantages over existing artificial muscles in that they feature a voltage-controlled linear contraction without the need for rigid components. Peano-HASELs have been shown to achieve muscle-like power densities of 160 W/kg and are capable of self-sensing their deformation via built-in capacitive sensing (Kellaris et al., 2018). In contrast to soft fluidic actuators, Peano-HASELs locally redistribute a hydraulic fluid, which reduces viscous losses [HASEL actuators have shown full-cycle efficiencies of 20% (Acome et al., 2018; Mitchell et al., 2019)] and enables high-speed operation with cut-off frequencies above 50 Hz and strain rates over 800%/s (Kellaris et al., 2018).

To date, the fundamental benefits and drawbacks of using electrohydraulic Peano-HASEL actuators to drive prosthetic fingers have not been identified by a systematic experimental study that compares performance to traditionally used DC motor actuators. This work describes a first attempt to integrate a Peano-HASEL actuator into a prosthetic device with the goal of informing future development of multifunctional prosthetic hands. A direct comparison between an existing Peano-HASEL and a commercially available DC motor actuator is presented. A kinematic model of the prosthetic finger is developed and used to modify the four-bar linkage used in the prosthetic finger design to better suit the characteristics of the Peano-HASEL actuators. An analytical model of the Peano-HASEL is then used to inform design modifications to the Peano-HASEL actuators in order to improve the force output while maintaining similar weight. The result is a system with promising force output over a wide range of flexion angles that is capable of both controllable and rapid response (Supplementary Video 1). We discuss the significance of the unique properties of Peano-HASELs in the context of prosthetic limb design.

## Materials and Methods

### Commercially Available Prosthetic Hand and DC Motor

The Bebionic v2 Prosthetic Hand (RSL Steeper) is a widely available multi-functional prosthetic hand (2011). The hand has five degrees-of-freedom (DoF, individual actuators for each digit and thumb) and a passively positionable thumb abduction joint. Each digit is underactuated, meaning a single input controls the position of both the metacarpal phalangeal (MCP) joint and the proximal interphalangeal (PIP) joint through a four-bar linkage. A linear displacement of the pin in a slot in the proximal phalange causes a coupled flexion of the MCP and PIP joints. The finger is compliant at both the MCP and PIP joints in that external loads can collapse the digit in flexion. A torsional spring is located in the PIP joint to cause passive extension while the pin within the slot is pulled by the actuator to cause powered flexion [see Belter et al. (2013) for further discussion].

The actuator for each digit in the Bebionic consists of a DC motor, a planetary gearhead, a spur gear train, and a lead screw. The motor is a 1,524 Faulhaber DC motor with a 14:1 plantary gearhead and an IE2-16 motor encoder. The output of the planetary transmission is a plastic spur gear pair from Reliance Precision Mechatronics. This linear drive causes a nut to translate vertically and interfaces with the digit through the pin in the curved slot. The linear drive is a non-backdrivable element, ensuring that electric power can be turned off while maintaining a stable grasp. As used, the DC motor and necessary transmission components weigh a total of 37 grams. The DC motor itself weighs 18 grams; the array of transmission components make up roughly 50% of the entire weight of the actuating system.

The Bebionic v2 Prosthetic Hand was chosen as a basis for this design effort because it is considered a standard of care device in clinical care of people with upper limb amputation. The Bebionic contains well-founded electromechanical design elements including motors for each digit, kinematic linkages to couple multiple joints together, and the production of clinically viable forces and speeds from each digit. These standards of prosthetic hand design serve as a baseline to make comparisons and draw conclusions on the fundamental trade-offs when using the Peano-HASEL actuators instead of DC motor actuators.

### Design and Fabrication of Peano-HASEL Actuators

A thorough description of the Peano-HASEL actuator is provided in Kellaris et al. (2018) and basic operating principles are depicted in Figure 2A. Here we briefly describe the actuator designs used in this experimental work. We refer to multiple Peano-HASEL actuators combined in parallel as stacks; two types of actuator stacks were manufactured for this work. The first stack consisted of four Peano-HASEL actuators, each with six pouches in series. Each pouch was 5 cm wide by 2 cm high, and the electrodes were 5 cm wide by 1 cm high. Eighteen-micrometer-thick biaxially oriented polypropylene (BOPP) film (70 gauge, 5020 film, Multi-Plastics) was used as the dielectric shell for this set of actuators. The second stack consisted of seven Peano-HASEL actuators, each with 12 pouches in series as seen in Figures 2B,C. Each pouch was 4 cm wide by 1 cm high, and the electrodes were 4 cm wide by 0.5 cm high. 12-μm-thick Mylar film (Mylar 850H, DuPont Teijin) was used for this set of actuators.

The Mylar actuators were designed for improved force output vs. the BOPP stack, based on previous work by Kellaris et al. (2019) as described in detail in Sections Kinematic Model for Prosthetic Finger/Actuator System and Improving Actuator Force Output. The pouches were fabricated following the process described in previous work (Mitchell et al., 2019). Both sets of actuators used conductive carbon ink for the electrodes (CI-2051, Engineering Materials Systems, Inc) and were filled with a dielectric transformer oil (Cargill Envirotemp FR3). Both stacks were mounted to laser-cut 0.125-mm-thick FR-4 fiberglass mounts using adhesive transfer tape (3M 924 tape). On the end closest to the high voltage leads, 0.001” thick Kapton tape (Dupont) was placed in between dielectric film layers in the region contained by the FR-4 fiberglass mounts based on observations that an additional insulating layer decreased the likelihood of dielectric breakdown in that region. These ancillary components are depicted in Figure 2B. The stacks were designed to replace the DC motor and entire transmission system; therefore, the weight of the stacks plus ancillary components represent the weight of the system it was designed to replace. The BOPP actuator system weighed 43.6 grams while the Mylar version weighed 38.8 grams; this weight includes all components necessary for mounting and connecting the stacks to the prosthetic finger. The difference in weight between the Peano-HASEL stacks and the original DC motor system represented the closest possible weight match using existing Peano-HASEL architecture.

### Kinematic Model for Prosthetic Finger/Actuator System

**Figure 3:**
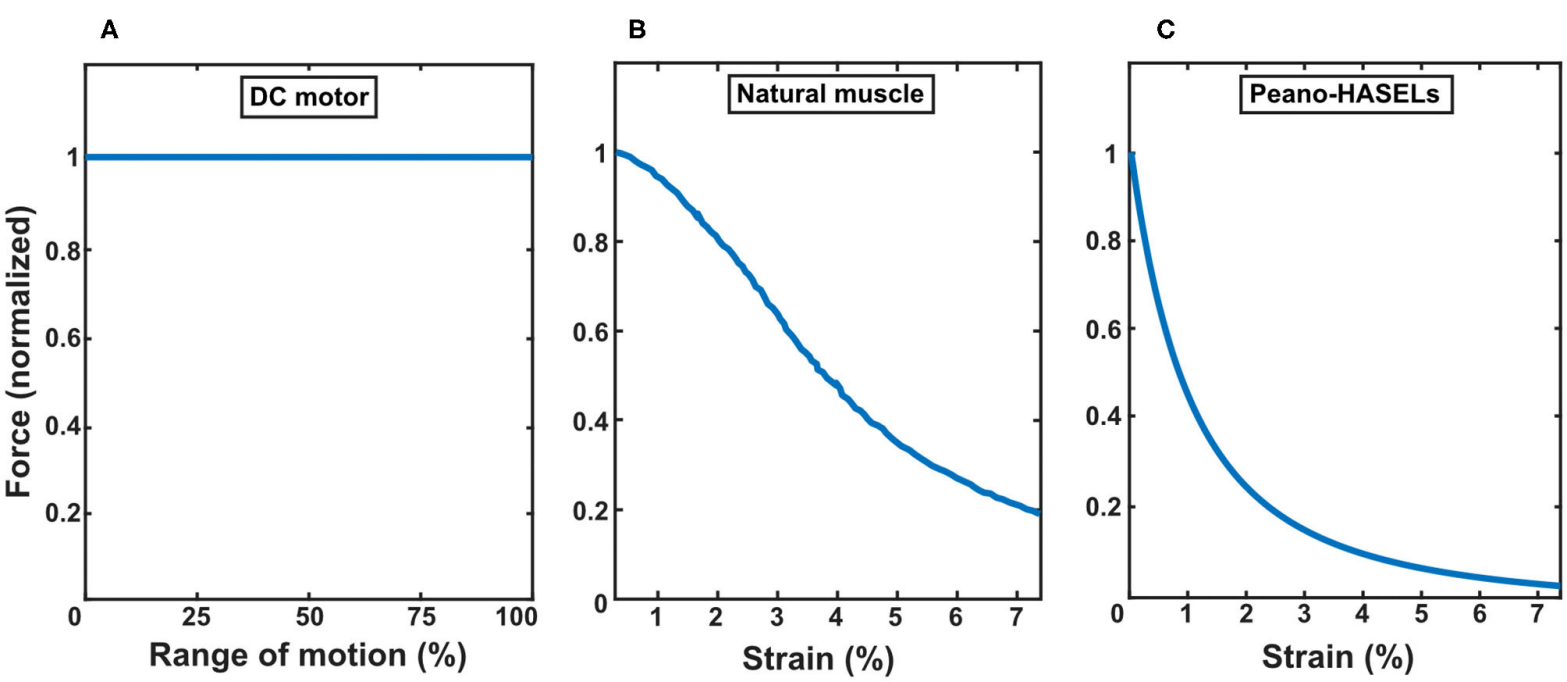
Comparing characteristics of force-displacement curves for three types of actuators: **(A)** DC motor, **(B)** natural muscle [derived from a study of tuna muscle (Shadwick and Syme, 2008)], and **(C)** Peano-HASELs. The force profile of each actuator is normalized to its blocked force. The Peano-HASEL actuators demonstrate behavior more closely resembling natural muscle than the DC motor.

Peano-HASELs provide a high blocking force that decreases over their linear stroke similar to the behavior of natural muscle. This behavior is unlike DC motors which produce torque that is proportional to the current provided and independent of the rotational position of the actuator output. A comparison of the force output of these three types of actuators can be seen in Figure 3 (Shadwick and Syme, 2008). The significant difference in actuator behavior drives the desire for an accurate model of the finger/actuator system that will allow us to study the changes in system behavior as various parameters, such as actuator force output, are modified. To appropriately integrate Peano-HASEL actuators with the prosthetic finger system, we developed a kinematic model to describe the prosthetic finger and actuator system.

**Figure 4:**
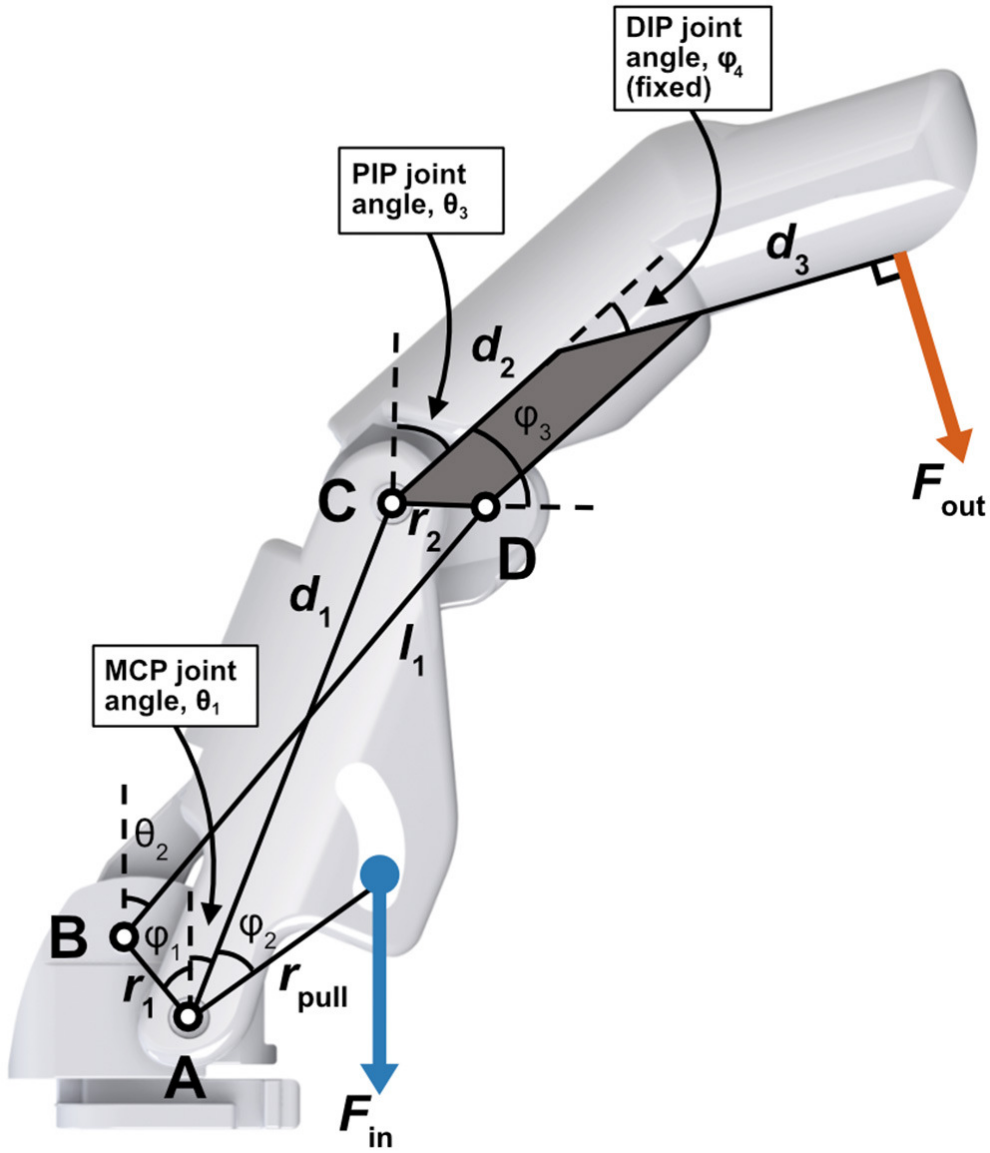
Schematic describing the prosthetic finger (based off of commercially available prosthetic hand) represented by a crank-rocker four-bar linkage as used in the kinematic model (Murali et al., 2019). The proximal and distal phalanges are fused, causing the distal interphalangeal (DIP) joint to be fixed, while the metacarpal phalangeal (MCP) and proximal interphalangeal (PIP) joints are free to rotate. Actuators apply load *F*_*in*_ at *r*_*pull*_. The pinch force is taken perpendicular to *d*_3_ and is represented by *F*_*out*_.

A kinematic system of an eight-bar linkage, which was derived by Murali et al. (2019) was adapted in this work to suit the four-bar linkage system used in the Bebionic v2 Prosthetic Hand (RSL Steeper). Figure 4 shows a crank-rocker system that is composed of binary linkages *r*_1_, *d*_1_, *l*_1_, *r*_2_, *d*_2_, *d*_3_ (Norton, 2000). *r*_*pull*_ represents the location from which the actuators (DC motor or Peano-HASELs) pull. φ_1_, φ_2_, φ_3_, φ_4_ are fixed, meaning they do not change value throughout flexion.

**Figure 5:**
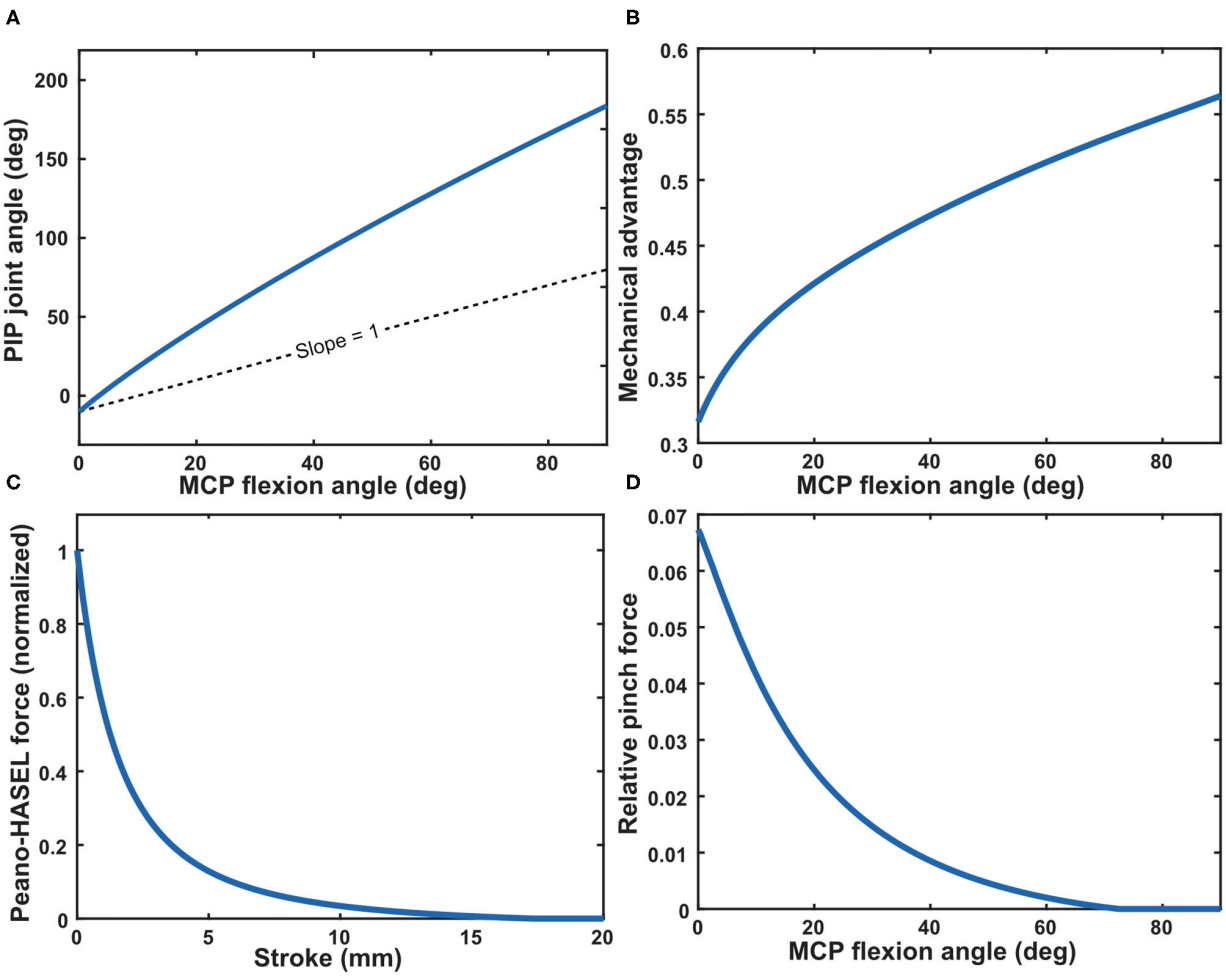
**(A)** Model prediction of the PIP joint angle as a function of the MCP joint angle through its flexion range (0–90°). **(B)** Mechanical advantage over MCP joint flexion range. In this case, the mechanical advantage is determined by the inverse of the slope of the PIP joint angle with respect to the MCP joint angle as shown in **(A)**. The mechanical advantage is equivalent to the ratio of torque about the PIP joint to torque about the MCP joint (Norton, 2000). **(C)** Force output for Peano-HASEL actuators as predicted by the analytical model (Kellaris et al., 2019), normalized by the theoretical blocking force of the actuator. **(D)** The mechanical advantage **(B)** of the kinematic system of the finger translates the force of the Peano-HASEL actuator **(C)** to the fingertip, resulting in the predicted fingertip pinch force **(D)**. The pinch force is depicted relative to the blocking force of the actuator.

The full kinematic model was developed using several equations derived by Norton (2000) and, in context of prosthetic finger design, in Murali et al. (2019). Figure 5 details how various parameters of interest behave over the MCP joint (θ_1_) flexion range which is constrained from 0 to 90°. The following derivation explains how these parameters of interest were determined.

First, link length *l*_1_ is obtained based on known initial conditions using Equation 1 (Murali et al., 2019).

(1) $$\begin{array}{l} l_{1}=\sqrt{{\left(d_{1}\text{ cos }\left(\theta_{1}\right)+r_{2}\text{ cos }\left(\theta_{3}+\varphi_{3}\right)-r_{1}\text{ cos }\left(\varphi_{1}\right)\right)}^{2}+{\left(d_{1}\text{ sin }\left(\theta_{1}\right)+r_{2}\text{ sin }\left(\theta_{3}+\varphi_{3}\right)-r_{1}\text{ sin }\left(\varphi_{1}\right)\right)}^{2}} \end{array}$$

Next, various joint angles are derived. We utilized Equation 2 to determine the behavior of the PIP joint angle θ_3_ with respect to the MCP joint angle θ_1_ as it changes linearly over its flexion range. This is shown in Figure 5A.

(2) $$\begin{array}{l} \theta_{3}=2\text{ arctan}\frac{-B\;\pm \;\sqrt{A^{2}+B^{2}-C^{2}}}{C-A}-\varphi_{3} \end{array}$$

Where

$$\begin{array}{l} \;A=2d_{1}r_{2}\text{ cos }\theta_{1}-2r_{1}r_{2}\text{ cos }\varphi_{1}\\ \;B=2d_{1}r_{2}\text{ sin }\theta_{1}-2r_{1}r_{2}\text{ sin }\varphi_{1}\\ C=d_{1}^{2}+r_{1}^{2}+r_{2}^{2}-l_{1}^{2}-2d_{1}r_{1}\left(\text{cos }\varphi_{1}\text{ cos }\theta_{1}+\text{ sin }\varphi_{1}\text{ sin }\theta_{1}\right) \end{array}$$

Finally, we use Equation 3 to find joint angle θ_2_.

(3) $$\begin{array}{l} \theta_{2}=2\text{ arctan}\frac{d_{1}\text{ sin }\left(\theta_{1}\right)+r_{2}\text{ sin }\left(\theta_{3}+\varphi_{3}\right)-r_{1}\text{ sin }\left(\varphi_{1}\right)}{d_{1}\text{ cos }\left(\theta_{1}\right)+r_{2}\text{ cos }\left(\theta_{3}+\varphi_{3}\right)-r_{1}\text{ cos }\left(\varphi_{1}\;\right)} \end{array}$$

We can now describe the theoretical orientation of the finger at any instance throughout its flexion range and subsequently the mechanical advantage of the system. Mechanical advantage *N* is used to determine how force output will translate from the actuators to the fingertip (Norton, 2000). This value is found by comparing the angular velocities ω of two points of interest, which is inversely proportional to the ratio of torque *T* about those same points (Norton, 2000). The mechanical advantage of point C with respect to point A at each instance over its flexion range is described in Equation 4 (Norton, 2000; Murali et al., 2019) and is plotted in Figure 5B.

(4) $$N=\frac{T_{C}}{T_{A}}=\frac{\omega_{A}}{\omega_{C}}=\frac{-r_{2}\text{ sin }(\theta_{2}-(\theta_{3}+\varphi_{3}))}{d_{1}\text{ sin }(\theta_{2}-\theta_{1})}$$

**Figure 6:**
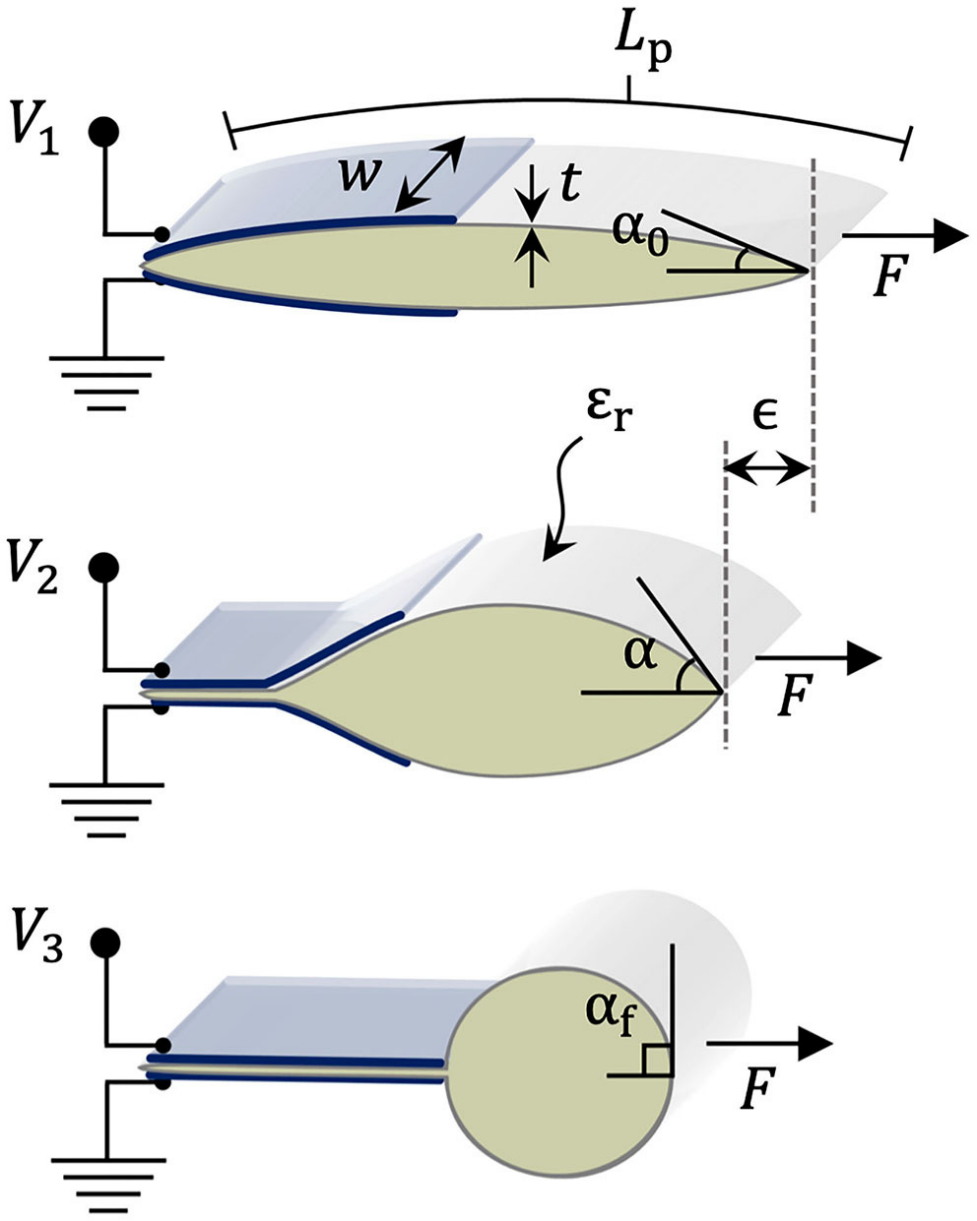
Schematic describing geometric and material parameters for Peano-HASEL actuators at different stages of the actuation process, as voltage increases from *V*_1_ to *V*_3_ (Kellaris et al., 2019).

Next, we integrated a previously developed analytical model for force output as a function of stroke of the Peano-HASEL actuators (Kellaris et al., 2019). We used this as the force input to the system, which allowed us to predict the theoretical force output at the fingertip when using Peano-HASELs as the actuation method, rather than the DC motor. Equation 5 describes the predicted force output of the Peano-HASEL actuators while Equation 6 predicts the corresponding stroke, represented by ϵ. Both are used to arrive at the force-stroke curve, presented with respect to MCP flexion angle, as seen in Figure 5C. The physical parameter represented by each variable can be seen in Figure 6.

(5) $$\begin{array}{l} F=\frac{w}{4t}\frac{\text{ cos }\left(\alpha \right)}{1-\text{ cos }\left(\alpha \right)}\varepsilon_{0}\varepsilon_{r}V^{2} \end{array}$$

(6) $$\begin{array}{l} \epsilon =1-\frac{\alpha_{0}}{\text{ sin }\left(\alpha_{0}\right)}\left(1+\frac{\sqrt{2A}}{L_{p}}\frac{\text{ sin }\left(\alpha \right)-\alpha }{\sqrt{\alpha -\text{ sin }\left(\alpha \right)\text{ cos }\left(\alpha \right)}}\;\right) \end{array}$$

With the above, we can next obtain the force output of the fingertip over the MCP flexion range. We calculated the torque input to the system by taking the cross product of the actuator force vector and the moment arm about point A (*r*_*pull*_). We multiplied this torque value with the mechanical advantage *N* to translate from torque about point A to torque about point C.

$$\begin{array}{l} T_{4}=N^{*}T_{2} \end{array}$$

Finally, we geometrically translated the torque about point C to the normal (pinch) force out at the fingertip *F*_*out*_ using Equation 7. The predicted pinch force can be seen in Figure 5D.

(7) $$\begin{array}{l} F_{out}=\frac{T_{4}}{|\vec{T_{Tip}}|\text{ cos }\left(\frac{d_{2}\text{ sin }\left(\theta_{3}+\varphi_{3}+\varphi_{4}\right)}{\left|\vec{T_{Tip}}\right|}\;\right)} \end{array}$$

Where $\vec{T_{Tip}}$ is the position vector of the fingertip as measured from point C, as shown in Equation 8.

(8) $$\begin{array}{l} \vec{T_{Tip}}=\left(d_{2}\text{ cos }\left(\theta_{3}\right)+d_{3}\text{ cos }\left(\theta_{3}+\varphi_{4}\right)\right)î\\ \begin{array}{l} \;+\left(d_{2}\text{ sin }\left(\theta_{3}\right)+d_{3}\text{ sin }\left(\theta_{3}+\varphi_{4}\right)\;\right)ĵ \end{array} \end{array}$$

Combining the full kinematic finger model with the Peano-HASEL model allows us to systematically alter finger parameters to change finger performance. This will be discussed further in the results section below.

### Experimental Setup and Protocol

To compare the DC motor actuator and Peano-HASEL actuators, as well as to validate model predictions, testing was performed over a series of experimental protocols.

#### General Experimental Design

Form factor measurements were recorded using hand-held calipers and a bench-top scale (Optima Scales OPK-S500). The Peano-HASEL was powered by a voltage signal with reversing polarity (Kellaris et al., 2018). A custom Matlab script sent the signal to a NI DAQ (Model USB6212), which interfaced with a high voltage amplifier (TREK 50/12). The TREK was limited to 6 mA maximum current. An 8-kV signal was used for the BOPP actuators, while 6 kV was used for the Mylar actuators based upon previously determined safe operating voltages for each material. The DC motor actuator was powered by a benchtop DC power supply at 9 V, and the current limit was set at 5 A. A custom LabView interface controlled the motor through a custom motor controller board (Sigenics Inc, Chicago IL). The current and voltage input to the DC motor actuator was monitored from the LCD readout of the desktop power supply (Keysight U8002A) to measure the electrical power consumption during experimental trials.

For all experiments, the prosthetic digit was mounted on an acrylic plate supported by 4 threaded rods. Two holes were laser-cut through the plate, one allowing the DC motor or Peano-HASELs to pull down and another to prevent the acrylic plate from interfering with flexion of the fingertip. For the Peano-HASEL actuators, the base of the stand included a mounting point to interface with the actuators and can be seen in Figure 1.

#### Characterization of Peano-HASEL Force-Stroke Curve

The force-stroke curves of the Peano-HASEL actuators were collected using a custom Matlab program that interfaced with both the TREK and an artificial muscle tester (Aurora Muscle Tester 310C-LR). The length of the actuators was recorded under a pretension of 40 N from the artificial muscle tester. From that point, the muscle tester arm moved in the direction of contraction in set increments, and the Peano-HASELs were actuated with a ramped square wave voltage signal 4 times at each increment. The average force was then calculated for each displacement step.

#### Force Measurement

For force measurements, a variable-position load cell (Phidget CZL616C) was mounted to the plate allowing for collection of fingertip force over a wide angular range. The load cell was calibrated using a set of calibration weights before the experimental protocol was completed. Using a SolidWorks model of the prosthetic finger, calibration blocks were 3D printed to ensure proper position of the load cell for each MCP angle of interest. The Peano-HASEL actuators were affixed in the test fixture as shown in Figure 1 and pre-tensioned by raising the height of the stand until the MCP angle reached 0°.

Force values were collected over two experimental protocols by varying the voltage signal sent to the TREK. To measure pinch force, the average pinch force value was collected over a 1 s grasp and repeated 5 times at each angle of interest. To measure continuous grasp, the force was collected over a 10 s grasp and repeated 4 times. Both tests included a 0.25 s ramp up and down during voltage transitions to minimize kinematic effects, though any spikes in force attributed to kinematics were ignored.

#### Power Consumption

A custom Matlab program acquired voltage data from the internal monitors of the Trek and current data from a current sensor (μCurrent GOLD) simultaneously with force testing to determine the energy consumption and power drawn during pinch and continuous grasp.

#### Dynamic Behavior

To measure free stroke, the load cell was removed and the finger was allowed to move freely through its maximum flexion range. The angle of the MCP joint was tracked optically (Canon EOS 6D DSLR) and measured using an open-source software program (Tracker version 5.1.3). The voltage signal was ramped over 0.5 s to minimize kinematic effects.

The dynamic specifications of the actuators were also tested. A high-speed camera (Model Phantom v710) was used to measure the temporal properties of the Peano-HASEL and a Canon EOS 6D DSLR was used with the DC motor actuator. The impulse speed was quantified using a square wave input signal at the appropriate voltage or pulse-width (100%) for the Peano-HASELs and DC motor, respectively. The bandwidth was studied by applying various frequencies between 0.5 and 50 Hz, using a reversing polarity sine wave at the appropriate voltage for the Peano-HASELs and a square-wave with 100% pulse-width for the DC motor. The angle of the MCP joint was tracked optically and processed, also using the software program Tracker. A resonant frequency in the Peano-HASEL actuator system was observed between 10 and 25 Hz. For those trials, small acrylic side constraints were added to minimize large motion perpendicular to the direction of actuation. For the Mylar stack of actuators, a 5.5 kV voltage signal was used (as opposed to the 6 kV amplitude for force testing), to reduce risk of electrical failure during the high cycling frequency required for dynamic testing.

During dynamic testing, no external loads were applied to the prosthetic finger system. However, the spring element (torsional spring for the original finger and elastic band for the custom finger) was present in the prosthetic finger system. This element provided a variable load proportional to the flexion magnitude which acted against the actuators, therefore all force and dynamic data was collected under a variable load condition.

## Experimental Results

### Validation of Kinematic Model

The original design, based on the commercially available Bebionic finger, was analyzed with the intention of validating the kinematic model. The torsional spring was removed in order to isolate the kinematic behavior of the finger. Without the torsional spring, the finger rested on the load cell at various angles. Force data was collected based off the described protocol at a range of MCP flexion angles.

**Figure 7:**
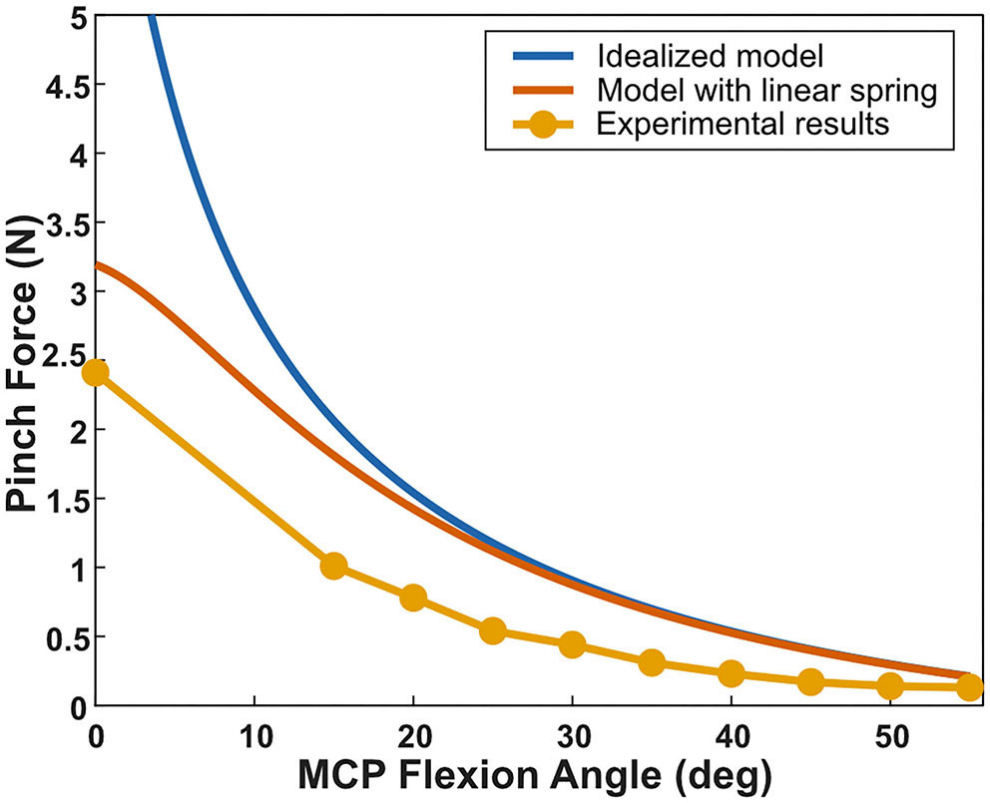
Comparing the experimental results for the fingertip pinch force as a function of MCP angle with the idealized model and the model that includes a linear spring. The linear spring constant was measured experimentally and accounts for unwanted deformation in both the prosthetic digit and the test setup as well as the elasticity of the Peano-HASEL actuators. The experimental results for the pinch force match well with the model that includes the linear spring.

The finger and test stand demonstrated elastic behavior during testing. In the finger, imperfect rotational joints contributed to unwanted deflection, and the thermoplastic used to 3D print the finger bent under load. The acrylic stand and load cell mount also deflected under load. This unwanted elastic behavior was modeled as a linear spring between the actuators and the prosthetic digit. The software program Tracker was used to measure the elasticity of the system- at a known force, the deflection of the fingertip was measured, allowing an estimate for the spring constant of the system to be obtained. As shown in Figure 7, when we incorporated this experimentally-measured spring constant in the system, we found that the model matched the experimental data very well.

While configuring the finger without the torsional spring is useful for validating the kinematic model, removing the torsional spring results in a prosthetic finger with no restoring force. In the original finger design that includes the torsional spring, the Peano-HASEL actuators did not provide any grip force past an MCP angle of 30° which is insufficient for gripping objects.

### Improving Actuator Force Output

**Figure 8:**
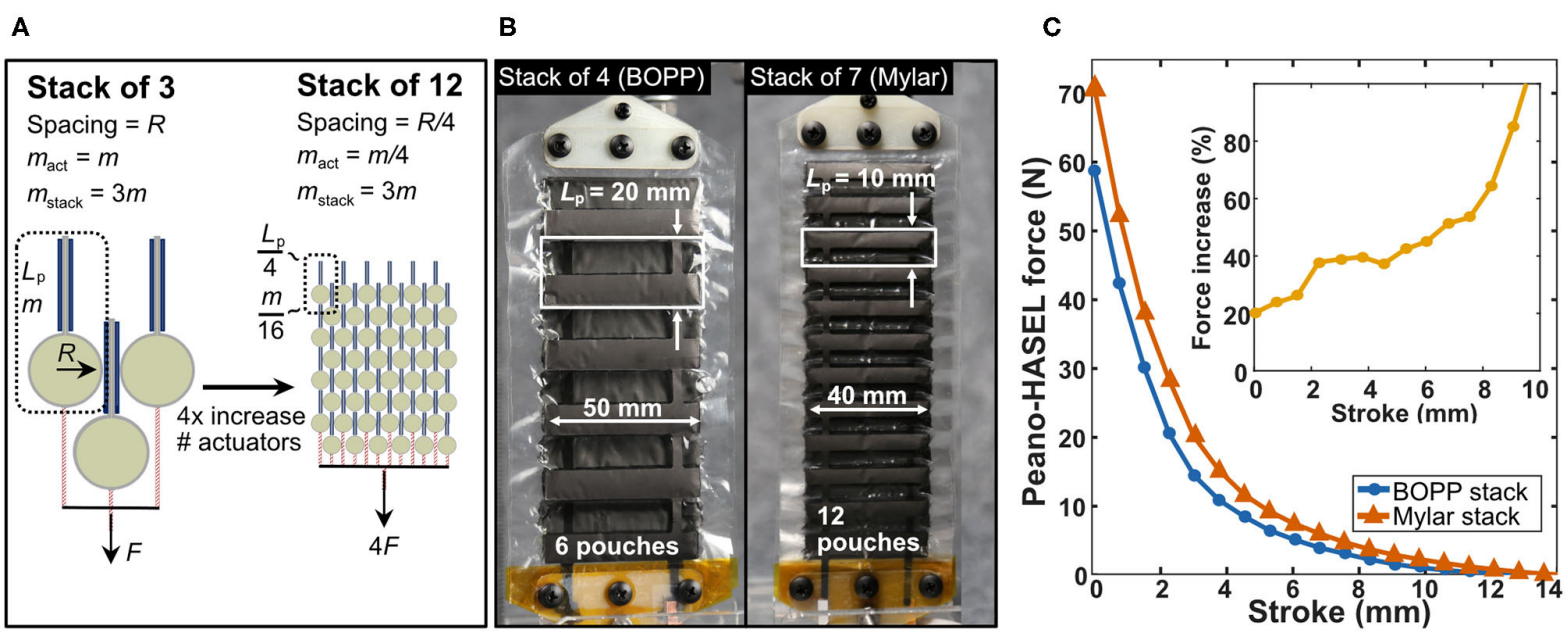
Designing weight-matched stacks of Peano-HASEL actuators with higher force output. **(A)** By fabricating actuators with decreased pouch length *L*_*p*_ and using a series of smaller pouches in place of a single larger pouch, the mass of the actuator is reduced without affecting its force-strain performance (Kellaris et al., 2019). In this way, more actuators can be stacked in parallel to produce a weight-matched stack with increased force output. **(B)** Photos of the actuators used in this work. The first stack of actuators tested used 4 actuators made from BOPP with 6 pouches each (43.6 g total). A second stack was produced with decreased pouch length using 7 actuators made from Mylar with 12 pouches each (38.8 g total). **(C)** Force-stroke curves for both stacks of Peano-HASEL actuators. The stack of Mylar Peano-HASEL actuators with shorter pouch length produced higher force than the original design while maintaining similar weight. The inset shows the relative force increase of the Mylar Peano-HASELs with respect to the BOPP Peano-HASELs.

First, we attempted to increase the force output of the finger by improving the force output from the Peano-HASEL actuator stack. In Equation 5, we observe that the force output *F* of a Peano-HASEL is independent of its pouch length *L*_*p*_. As a result, Kellaris et al. showed that actuators comprised of a series of shorter pouches have a smaller overall mass *m*_*act*_ than actuators comprised of fewer but longer pouches, while maintaining the same force-stroke characteristics (Figure 8A; Kellaris et al., 2019); thus, fabricating actuators with shorter pouches allows us to stack more actuators in parallel, while maintaining the same weight for the stack overall.

Following this strategy, actuators with decreased pouch length were constructed from Mylar film, with pouch geometries shown in Figure 8B. The reduced weight of these actuators allowed seven Mylar actuators to be stacked in parallel for improved force output while maintaining the same overall weight as the stack of four BOPP actuators. This film also has a higher permittivity, which acts to increase force output, according to Equation 5. Mylar film with a thickness of 12-μm was used, rather than the 18-μm BOPP, to help minimize losses due to bending stiffness of the film (Kellaris et al., 2019) and to reduce necessary operating voltages. The width of the pouches was decreased from 5 to 4 cm to reduce instabilities in actuation, as reported by Rothemund et al. (2019). While decreasing film thickness and pouch width act to lower the force output according to Equation 5, the larger number of actuators and the higher permittivity film offset these effects and increased the overall force output of the stack. Figure 8C shows the experimental force-stroke curves for the BOPP stack of four and the Mylar stack of seven, demonstrating the improved force output for the Mylar actuator stack.

### Improvement of Finger Design

A second way to improve the force output of the prosthetic finger is to modify the finger kinematic design for actuation with Peano-HASELs rather than a DC motor. The previously discussed kinematic model was used to evaluate how modifications to the four-bar linkage finger system would affect the fingertip force output at various grip angles when actuated with Peano-HASELs.

The Peano-HASELs provided forces up to 2.57 N at very low flexion angles (0–2°), but many gripping tasks fall within a range of 24–55° (Lee and Jung, 2016). Parameters were systematically changed with the goal of increasing force output within this common grip range. Without additional constraints many finger designs resulted in non-lifelike actuation or behavior very different from the original design, based on the Bebionic finger (e.g., no flexion in the PIP joint across the entire range of flexion in the MCP joint). Although these designs predicted higher force output in the desired range, they were deemed unacceptable as the prosthetic device must mimic the intact biological system it resembles.

Parameters were constrained such that (1) the relaxed position of the finger would not change, (2) the fingertip itself would be at a similar location in space as compared to the original design, when the MCP angle was flexed to 35° (ensuring appropriate flexion of the PIP joint), and (3) the proximal and distal phalanges' size and shape remained the same as the original design, maintaining the lifelike appearance of the finger. Under these conditions, new finger parameters were selected. The custom finger components were printed with a 3D printer (Formlabs Form 2). At each joint two members were connected with a metal pin, with one member of each joint designed for a press-fit while the other member was designed for a freely rotating fit.

We also removed the torsional spring in the custom finger design and replaced it with an elastic band (1/4″ diameter 2.5 oz force, Prairie Horse Supply) spanning from the knuckle joint to the proximal phalanx on the dorsal side of the finger. The elastic band was added to serve as a restoring force which pulled the finger back to its resting position.

**Figure 9:**
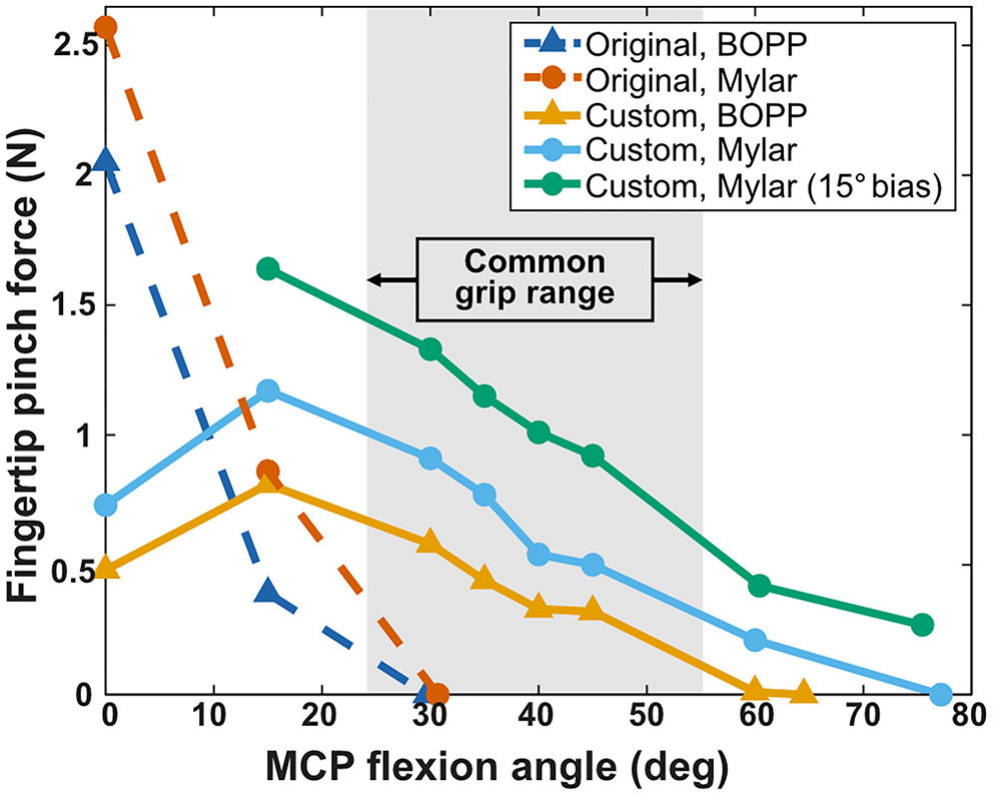
Fingertip pinch force across original and custom prosthetic finger designs and BOPP and Mylar stacks of Peano-HASEL actuators. The original finger (dashed lines) provides a high pinch force that rapidly decreases, providing no pinch force over 30°. The custom prosthetic finger sacrifices higher blocked force for increased pinch force within the common grip range (24–55°) (Lee and Jung, 2016) and provides over twice the flexion range as the original design. The resting MCP angle of a human finger can be over 30°, so an initial bias of 15° was applied to further increase the fingertip force within the common grip range (Lee et al., 2008; Lee and Jung, 2016).

The results of force vs. MCP angle are presented for the original and custom finger designs in Figure 9. For each design, force data was collected using both the BOPP and Mylar Peano-HASELs. In all cases, the Mylar Peano-HASELs increased the fingertip force output as compared to the BOPP Peano-HASELs, with an average increase of 62%. At a blocked finger position (0°) the original finger design provides the highest force, but decreases rapidly to its limit at ~30°. The custom finger design sacrifices pinch force at the lowest angles for drastically increased fingertip force within a common grip range (24–55°) (Lee and Jung, 2016). We further increased the pinch force by an average 68% within this range by adding an initial bias of 15°. This accounted for the fact that the MCP angle of a relaxed human finger can be over 30° (Lee et al., 2008; Lee and Jung, 2014).

The custom finger also greatly increased the range of motion of the prosthetic finger. Various experiments have indicated the natural maximum angle of MCP flexion is between 60 and 90° (Lee and Jung, 2014). While the original design provided no pinch force past 30°, the custom finger (without initial bias) reached 77° of flexion.

### Further Kinematic Characterization

#### Energy Consumption in Pinch

**Figure 10:**
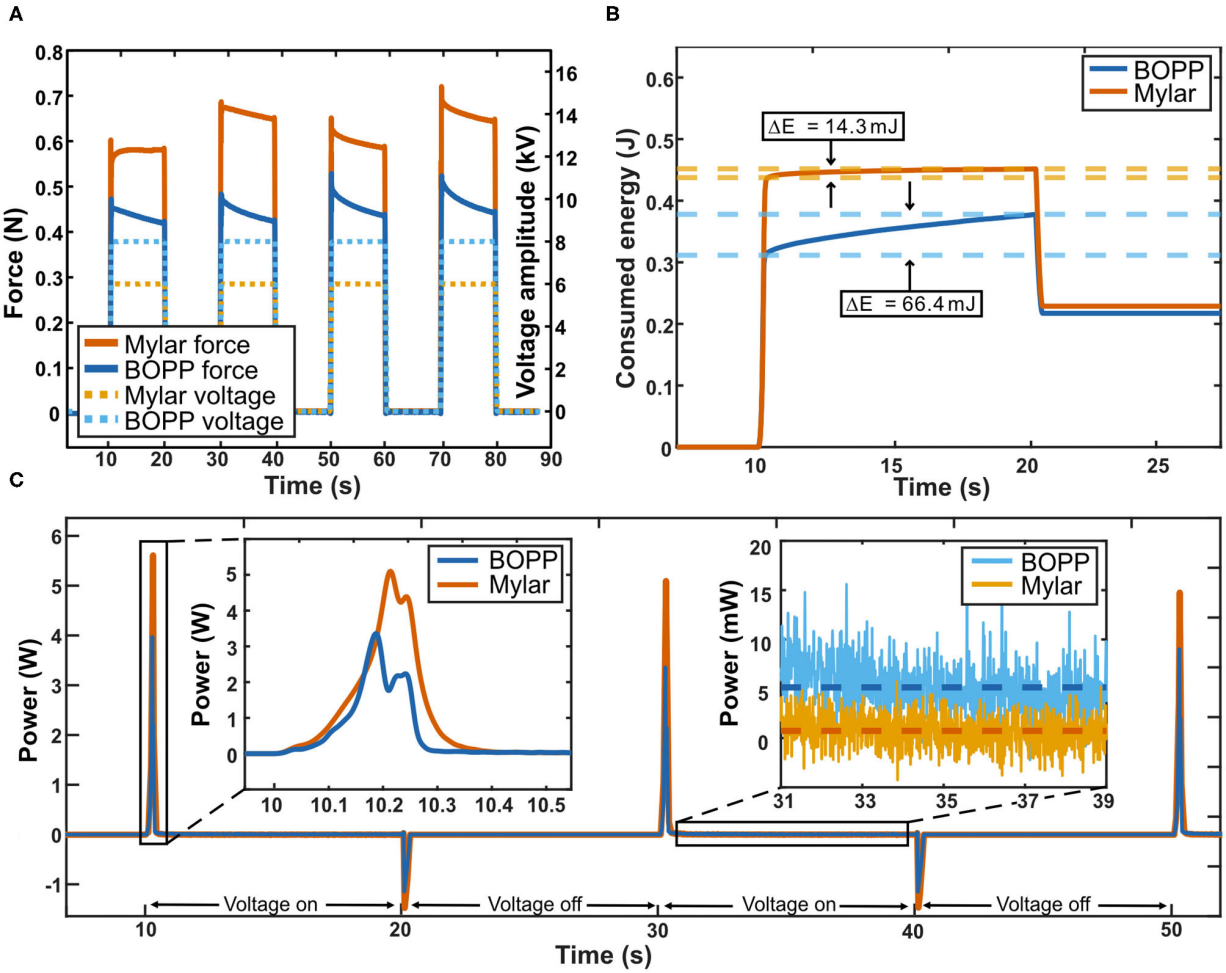
Characterizing energy consumption of Peano-HASEL actuators during grasping. **(A)** Voltage signals (dashed line) applied for 4 repeated 10 s grasps. The corresponding pinch force is also shown (solid line). **(B)** Energy consumed during 10 s continuous grasp. Both BOPP and Mylar actuators used very little energy to maintain their state during continuous grasp due to their “catch” state. Mylar demonstrated lower energy consumption (14.3 mJ) during a continuous grasp than BOPP (66.4 mJ). During actuator relaxation, energy can be recovered via additional circuitry that harnesses the discharge current. **(C)** Power draw over multiple 10 s continuous grasp cycles. Power drawn by the actuators over the 10 s continuous grasp was largely contained to the 0.25 s voltage ramp causing initial flexion of the finger (inset, left). During continuous grasp the BOPP Peano-HASELs drew an average of 5.6 mW while the Mylar Peano-HASELs drew an average of just 0.94 mW (inset, right).

Driving a prosthetic finger with Peano-HASELs provides many additional benefits that are relevant to the user. We characterized energy consumption behavior based on the previously described protocol for grip and 10 s hold. The applied voltage and corresponding force output across four testing cycles can be seen in Figure 10A. As seen in Figure 10B, the BOPP consumes 66.4 mJ over the 10 s hold, while Mylar consumes 14.3 mJ over the same hold. The DC motor actuation system does not require additional energy to hold its position, but only through the use of additional transmission components. Without those components, the DC motor requires 2,100 mJ to hold its position. The HASEL actuators do not require any additional components to display this behavior. This property—known as having a catch state—is typical for electrostatic actuator systems.

The power draw for both types of actuators was determined from the collected data and is presented in Figure 10C. The Mylar stack consisted of 7 actuators, compared to the 4 BOPP actuators, resulting in a higher power draw during flexion (Figure 10C, inset left). However, during continuous grip, the BOPP consumed an average of 5.6 mW while the Mylar consumes an average of just 0.94 mW. This is due to the lower leakage current the Mylar film displays. Overall, Peano-HASEL actuators consume power primarily during transitions between actuation states, with very little power consumption during the hold state.

### Dynamic Characterization of Actuators

The kinematic properties of the actuators include the range of motion of the prosthetic finger (in degrees), the maximum angular speed of the prosthetic finger (in degrees/second), and the bandwidth of the prosthetic finger system (in Hertz).

#### Stroke

The range of motion of the original prosthetic finger when actuated with the BOPP Peano-HASELs (29.7°) corresponded to 35% of the maximum range of motion when actuated with the DC motor (85°). With the custom finger design and Mylar Peano-HASELs, the range of motion increased to 77.17°, corresponding to 91% of the range of motion of the original design (DC motor system).

#### Step-Voltage Response

A major advantage of Peano-HASEL actuators is their use of a fast electrohydraulic mechanism (Kellaris et al., 2018). The dynamics of Peano-HASEL actuators under different loads and operating conditions were studied in detail by Rothemund et al. (2020). To highlight the fundamental benefits and drawbacks of the Peano-HASEL and DC motor actuators, the dynamics of the prosthetic finger system in this work were studied under no load.

**Figure 11:**
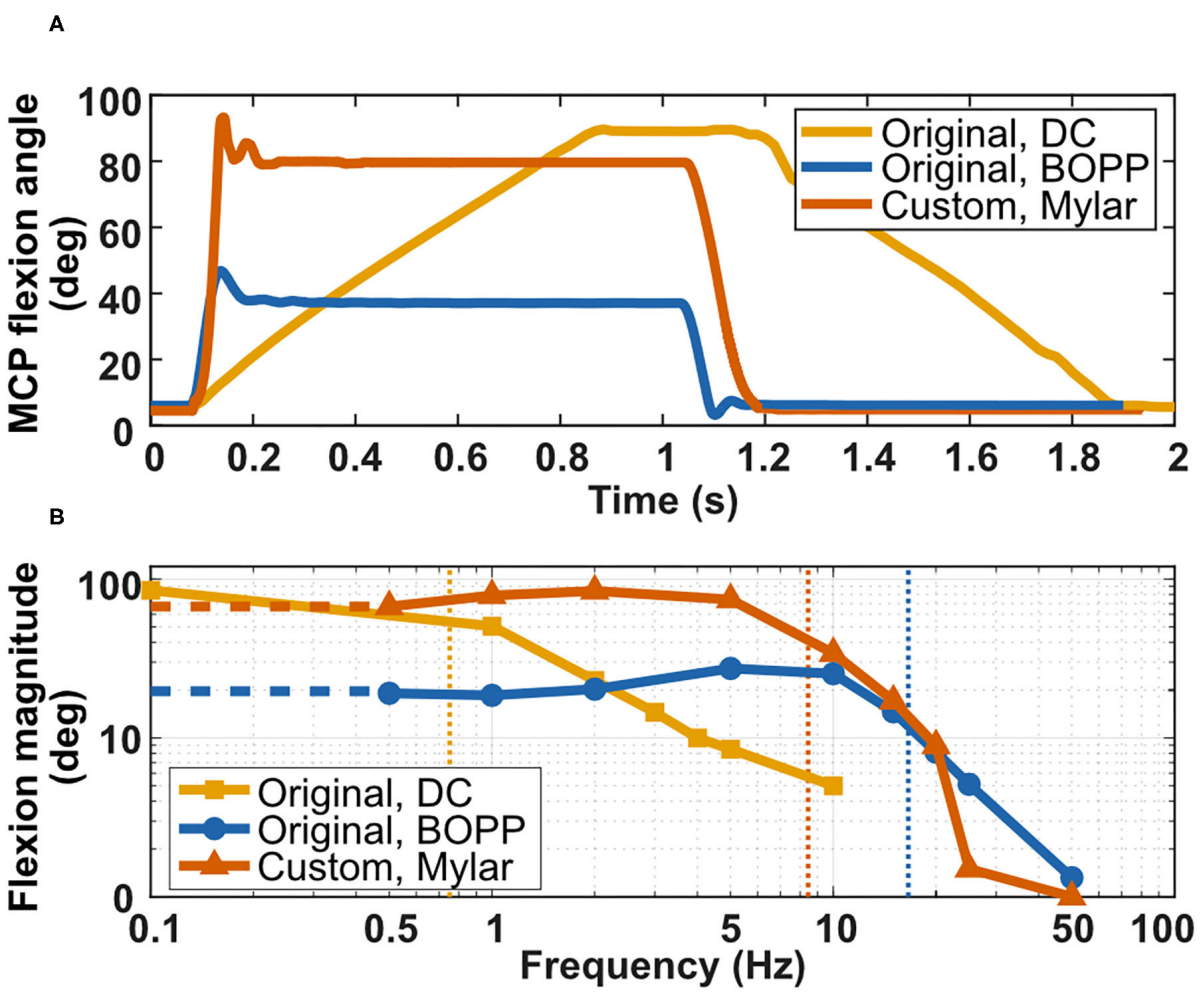
Characterizing the dynamic performance of the prosthetic fingers. **(A)** Comparing impulse response for (1) the original finger actuated by DC motor, (2) the original finger actuated by BOPP Peano-HASELs, and (3) the custom finger actuated by Mylar Peano-HASELs. **(B)** Bode plot comparing roll-off frequencies. The roll-off frequency was considered at −3 dB of the low-frequency amplitude.

Therefore, in our experiments the maximum flexion speed was measured about the axis of rotation at the MCP joint and is plotted in Figure 11A. The original finger actuated by BOPP Peano-HASELs demonstrated an average angular speed of flexion (738 deg/s) that was 4.9 times greater than the original finger design actuated by the DC motor actuator (150 deg/s). With the custom finger and Mylar Peano-HASELs, the average angular speed of flexion was 10.6 times greater (1,587 deg/s) than the original finger design actuated by the DC motor (Supplementary Video 1). The slope of the line in Figure 11A indicates the instantaneous angular velocity of the finger.

#### Frequency Response

Peano-HASEL actuators display a very high bandwidth (Kellaris et al., 2018). The frequency response of the system was characterized, with the cutoff frequency considered when the displacement is −3 dB of the maximum angular displacement. The bandwidth of the original finger when actuated by the BOPP Peano-HASELs is 21 times greater (15.9 Hz) than the original finger actuated by the DC motor (0.75 Hz), and the bandwidth for the custom finger actuated by Mylar Peano-HASELs was 11.1 times greater (8.3 Hz) than the original finger actuated by the DC motor. Supplementary Video 2 demonstrates actuation of this system at 1 and 5 Hz for a sinusoidal input voltage signal at 5.5 kV.

When the custom finger was actuated with the Mylar Peano-HASELs, there is a rapid decrease in amplitude that can be attributed to significant resonance in the actuator system from 10–25 Hz. Rather than actuating the finger, the Peano-HASELs moved side-to-side creating large oscillations perpendicular to the desired direction of actuation of the Peano-HASELs (Supplementary Video 3). Real-world applications of this technology would include mechanical constraints to prevent this out-of-plane behavior. The Bode plot for both systems can be seen in Figure 11B.

## Discussion and Outlook

This work is a first attempt to drive a prosthetic finger with Peano-HASEL actuators. Performance is compared to existing DC motor actuators used today. The benefits and drawbacks to this new actuator technology are elucidated by the results summarized in Table 1.

**Table 1:** Summary of collected data comparing the original finger design actuated by both the DC motor and BOPP Peano-HASELs with the custom finger actuated by Mylar Peano-HASELs.

	**Original finger design, DC motor**	**Original finger design, BOPP Peano-HASELs**	**Custom finger design, Mylar Peano-HASELs**
Actuator weight (g)	37.0	43.6	38.8
Length × width × depth (cm)	5 × 2 × 1.5	15.5 × 5.4 × 0.65	16 × 7 × 0.64
Volume (cm^3^)	15	54.4	71.7
Range of motion (deg)	85	29.7	77.17
Force at 30° (N)	13	0	0.91 (1.33)
Energy consumed to grasp (mJ)	3,800	311.4	437.5
Average power consumption during grasp (mW)	9,200^*^	5.6	0.94
Flexion speed (deg/s)	150	738	1,587
Bandwidth (Hz)	0.75	15.9	8.34
Flexion magnitude at 10 Hz (deg)	5.0	25.4	34.1

*Values in parentheses are those measured using a 15° offset in initial MCP angle*.

* *Assumes continuous current draw to maintain force during grasp when using DC motor actuator*.

The custom finger actuated by Peano-HASEL actuators provided many benefits as compared to the original finger design powered by the DC motor. The custom finger system is 10.6 times faster, has 11.1 times higher bandwidth, and consumes 8.7 times less electrical energy to grasp. It reaches 91% of the maximum range of motion of the original finger.

However, the force production of these Peano-HASELs is substantially less than the DC motor actuator (~10× decrease). Smaby et al. identified average pinch force values for every-day tasks which range from 1.4 N (push remote button) to 31.4 N (insert slippery plug into wall outlet) (Smaby et al., 2004). The current fingertip pinch force from Peano-HASEL actuators is still insufficient for many of these tasks. Two properties of the custom prosthetic finger system primarily affect this result: (1) specific energy of the Peano-HASEL actuators and (2) design of the prosthetic finger.

### Specific Energy of Peano-HASEL Actuators

The analytical model presented by Kellaris et al. (2019) details how we can produce a Peano-HASEL stack with higher specific energy that consequently results in higher force output while keeping the total weight constant. Production of these actuators is currently limited by available plastic films and manufacturing techniques. As the pouch size becomes smaller, the bending stiffness of the plastic plays a more prominent role in limiting actuation (Kellaris et al., 2019). In the future, Peano-HASEL stacks with a substantial increase in specific energy may be possible using thinner plastic films and borrowing multi-layer soft lithography or precision laser micromachining processes that have been shown to produce fluidic actuators on the 100-μm scale (Ho and Jow, 2009; Moretti et al., 2018). Pouches on these length scales would allow for actuator stacks with drastically improved force output. In addition to scaling down pouch lengths, driving voltage can be increased to further increase the specific energy of the Peano-HASEL actuators. The force production of the Peano-HASEL is proportional to the square of the excitation voltage, so finding a reliable way to increase the excitation voltage without damaging the actuators (e.g., different materials, better manufacturing techniques) would cause a quadratic increase in force production (Suo, 2010; Acome et al., 2018; Kellaris et al., 2018).

### Design of the Prosthetic Finger System

Altering the kinematics of the prosthetic finger increased the fingertip force at common grip angles. However, there are still opportunities for further improvements to the kinematic design. The finger was changed under many constraints; modifying these constraints could lead to more design freedom allowing for higher force production at various angles. As manufactured, the custom prosthetic finger demonstrated elastic behavior. A desktop 3D printer was used with resolution set to 100 μm, and the metal pin joints were modified and assembled by hand. Better manufacturing techniques with stiffer materials would lead to less elastic behavior and less frictional losses in the system, resulting in higher fingertip force output (Ngo et al., 2018). In this work a four-bar linkage design was used, but other designs currently in use could be adapted and optimized for actuation with Peano-HASELs, including other four-bar linkage conformations (Vincent by Vincent Systems), tendon-roller systems (iLimb by Touch Bionics) and intrinsic finger actuation designs like those presented by Murali et al. (2019). Overall, the results presented here show the first steps toward improving upon the standard design of prosthetic devices and indicates that further research in inventing prosthetic mechanisms specifically designed for the Peano-HASEL actuators is warranted.

The dynamic performance of the Peano-HASELs exceeds the abilities of the DC motor actuator. With the custom finger, maximum speed of flexion was 10.6 times greater and the bandwidth was 11.1 times greater than the DC motor actuator. Reduced pouch dimensions and lower viscosity dielectric fluids could lead to even faster actuation (Rothemund et al., 2020). This dynamic performance will become even more important as improved myoelectric control algorithms become available for prosthetic hands. Furthermore, the lifetime of currently available Peano-HASEL actuators is less than that of well-established DC motors. Peano-HASELs produced over 20,000 cycles when operated at their upper voltage limit (Kellaris et al., 2018). Kellaris et al. modeled Peano-HASEL actuators that could maintain their force output while operating at lower voltages, thereby resulting in longer lifetimes (Kellaris et al., 2019).

There are several additional aspects of the Peano-HASEL actuators that make them attractive for further development toward practical implementation in upper-limb prosthetic design, including: (1) bio-mimetic form factor and weight distribution, (2) the series-elastic nature of the actuators, (3) their self-sensing capability, (4) their compatibility with novel myoelectric control systems, (5) the availability of miniaturized high-voltage power electronics.

### Bio-Mimetic Form Factor and Weight Distribution

**Figure 12:**
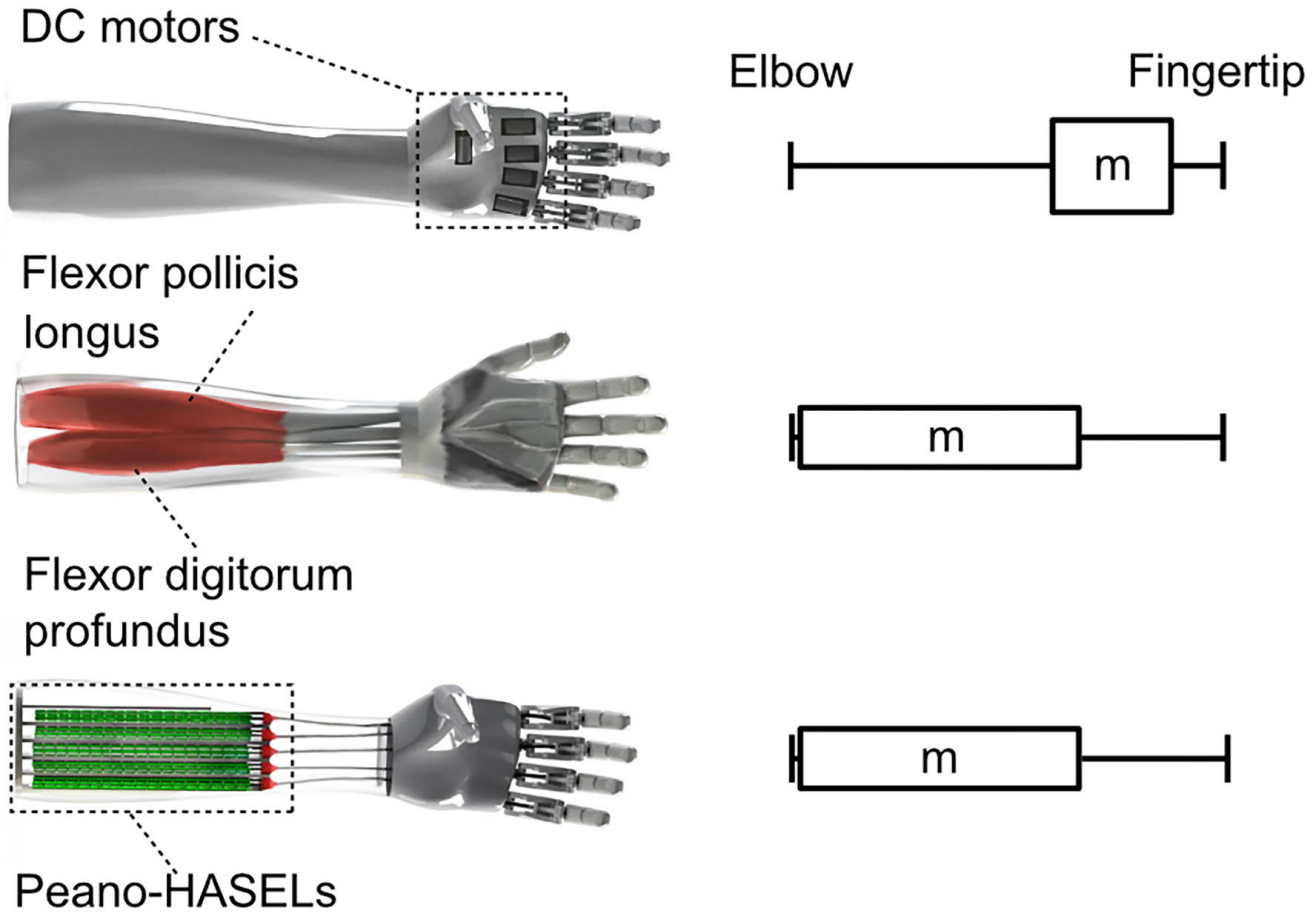
Peano-HASEL actuators simplify the design of prosthetic limbs that have a more proximal weight distribution, which decreases the load experienced by the user. The overall design of a prosthetic limb based on Peano-HASEL actuators can better mimic the physiology of the human arm.

The tradeoff between force and weight has plagued prosthetic device design, since prosthetic users consider weight to be one of the major deficits of upper-limb prosthetic devices today (Biddiss et al., 2007; Cordella et al., 2016). The distribution of weight in a prosthetic socket can have an outsized effect due to the moment arm between the residual limb and prosthetic hand. In effect, additional weight on the distal end is amplified by these large moment arms and promotes actuator design which can be distributed away from the distal end of the prosthesis. The flat form factor of Peano-HASEL actuators, coupled with their inherent linear contraction on activation—without the need for bulky gears and transmission systems—means they can more easily apply tension to a tendon/cable from afar. The actuators could then be located more proximally in the prosthetic socket which would more closely resemble the distribution of weight in an intact human limb (Figure 12). This design change would reduce the load experienced by the user and could lead to additional benefits like compliant digits/palm and robustness to environmental hazards like water, dirt, etc.

### Series-Elastic Nature of the Actuators

The series-elastic nature of Peano-HASEL actuators is based on compliance of the electrohydraulic structure while exposed to varying external loads. The benefit of a series elastic actuator in a prosthetic device includes improved robustness to perturbation and safety when interacting with people and/or delicate objects (Sensinger and Weir, 2008). Peano-HASELs would enable these features without any additional componentry (like springs, sensors, and controllers) typically required to create a series elastic actuator when using a DC motor.

### Self-Sensing Capability

The self-sensing nature of deformable capacitors stems from changes in capacitance during actuation (Acome et al., 2018), which can be mapped to the state of the actuator and used in a closed-loop position control system (Schunk et al., 2018; Ly et al., 2020). The ability of Peano-HASELs to inherently self-sense position could result in space/time/cost savings compared to DC motors, which require additional ancillary components in order to determine their rotational position in space (typically a motor encoder).

### Compatibility With Novel Myoelectric Control Systems

Myoelectric control systems are the most commonly used methodology to interface the amputee to the prosthetic limb (Childress and Weir, 2004). The measurement of muscle activity in the residual limb is used as a control signal to determine the position/speed of an actuator in the device. Significant research has focused on the best algorithms for this mapping (Scheme and Englehart, 2011). In all cases, the use of a Peano-HASEL can be incorporated into a myoelectric control system. As shown in this work, control signals can be sent from a controller to high-voltage power electronics to actuate the Peano-HASELs. Therefore, new myoelectric control algorithms could be developed that take advantage of the stacked design of Peano-HASELs, which mimic the structure of our intact muscular anatomy based on hierarchical muscle anatomy. The force that a muscle creates is dependent on the firing rate of the action potentials (similar to the excitation voltage of the Peano-HASEL) and the number of motor units that are recruited (similar to the number of pouches/actuators in the Peano-HASEL stack that are activated) (Enoka, 1995). The modulation of the number of Peano-HASEL pouches/actuators that are active at any given time permits soft, delicate motion as well as powerful grasps. A similar control methodology is not possible using established DC motor technology.

### Availability of Miniaturized High-Voltage Power Electronics

Finally, commercially available high-voltage amplifiers and switches are already available in small form factors which could be fitted inside typical prosthetic sockets (Schlatter et al., 2018). Supplementary Table 1 details such miniature electronics that could be used, with size, weight and voltage operating limits. Existing lithium ion batteries (FlexCell, Infinite Biomedical Technologies) could be used to power these circuits and actuate Peano-HASELs. The safety of the amputee is of concern when high-voltage components are used in prosthetic systems. Insulating materials such as rubber can be used to shield the user (Pourazadi et al., 2017), and actuator stacks can be designed such that the outermost electrodes in the stack are grounded, further shielding the user from high voltage to allow safe incorporation into a prosthetic limb.

This paper presents the remaining challenges and highlights the strong potential of Peano-HASEL actuators to realize the next generation of multi-functional and lifelike prosthetic devices.

## Data Availability Statement

The original contributions presented in the study are included in the article/Supplementary Materials, further inquiries can be directed to the corresponding author/s.

## Author Contributions

CK and JS conceived and supervised the research. ZY and NK conceived the design of experiments and fabricated actuators. ZY collected and analyzed data with help from CC-M. CC-M and ZY developed the kinematic model of the finger and actuator system with guidance from JS and NK. ZY, CC-M, and JS designed the prosthetic fingers used in this work. DR and CC-M designed the testing fixture for force output tests. MBE helped design early prototypes of the experimental setup. SKM and JS collected and analyzed DC motor data. ZY, NK, CC-M, and JS drafted and revised the manuscript and figures, with help from DR and guidance from CK. All authors contributed to the article and approved the submitted version.

## Conflict of Interest

NK, SKM, and CK are listed as inventors on patent applications PCT/US18/023797 and PCT/US19/020568 that cover fundamentals and basic designs of HASEL actuators. NK, SKM, and CK are co-founders of Artimus Robotics, a start-up company commercializing electrohydraulic HASEL actuators. The remaining authors declare that the research was conducted in the absence of any commercial or financial relationships that could be construed as a potential conflict of interest.
