# Supplementary material for: Design of a High-Speed Prosthetic Finger Driven by Peano-HASEL Actuators
Source: Front Robot AI. 2020 Nov 27;7:586216. doi: 10.3389/frobt.2020.586216 (PMC7805944; doi:10.3389/frobt.2020.586216)
Supplement: Supplementary file 4 [file Table_1.DOCX]

Supplementary Material

Table S1. Details on commercially available miniaturized high-voltage electronics that could readily be used for designing prosthetic hands driven by Peano-HASEL actuators.

| **Manufacturer** | **Part Number** | **Function** | **Maximum operating voltage** | **Dimensions (length x width x height in mm)** | **Weight (g)** |
| --- | --- | --- | --- | --- | --- |
| Pico Electronics | 5VV10 | Proportional high-voltage amplifier | 10 kV | 57.15 x 28.58 x 12.7 | 45 |
| XP Power | AG series | Proportional high-voltage amplifier | 3-5 kV | 28.7 x 17.53 x 6.35 | 7.09 |
| Voltage Multipliers Inc. | OC100HG | Optocoupler (high-voltage switch) | 10 kV | 25.4 x 11.4 x 8.13 | 4.5 |
| IXYS | IXTT02N450HV | N-channel enhancement mode MOSFET (high-voltage switch) | 4.5 kV | 14 x 16.05 x 5.1 | 4 |
